# Training-Dependent Change in Content of Association in Appetitive Pavlovian Conditioning

**DOI:** 10.3389/fnbeh.2021.750131

**Published:** 2021-11-25

**Authors:** Hea-jin Kim, Hae-Young Koh

**Affiliations:** ^1^Center for Neuroscience, Brain Science Institute, Korea Institute of Science and Technology (KIST), Seoul, South Korea; ^2^Division of Bio-Medical Science and Technology, KIST School, Korea University of Science and Technology, Seoul, South Korea

**Keywords:** mice, Pavlovian conditioning, association, mediated learning, reality testing, conditioned hallucination, mPFC, PLCβ1

## Abstract

In appetitive Pavlovian conditioning, experience with a conditional relationship between a cue [conditioned stimulus (CS)] and a reward [unconditioned stimulus (US)] bestows CS with the ability to promote adaptive behavior patterns. Different features of US (e.g., identity-specific sensory, general motivational) can be encoded by CS based on the nature of the CS-US relationship experienced (e.g., temporal factors such as training amount) and the content of association may determine the influence of CS over behavior (e.g., mediated learning, conditioned reinforcement). The content of association changed with varying conditioning factors, thereby altering behavioral consequences, however, has never been addressed in relevant brain signals evoked by CS. Our previous study found that phospholipase C β1-knockout (*PLC*β*1*-KO) mice display persistent mediated learning over the extended course of odor-sugar conditioning, and that wild-type (WT) mice lose mediated learning sensitivity after extended training. In this study, in order to see whether this behavioral difference between these two genotypes comes from a difference in the course of association content, we examined whether odor CS can evoke the taste sensory representation of an absent sugar US after minimal- and extended training in these mice. In contrast to WT, which lost CS-evoked neural activation (c-Fos expression) in the gustatory cortex after extended training, KO mice displayed persistent association with the sensory feature of sugar, suggesting that sensory encoding is reliably linked to mediated learning sensitivity and there is a training-dependent change in the content of association in WT. *PLC*β*1* knockdown in the left medial prefrontal cortex (mPFC) resulted in mediated learning sensitivity and CS-evoked gustatory cortical activation after extended training, proposing a molecular component of the neural system underlying this Pavlovian conditioning process. We also discuss how disruption of this process is implicated for hallucination-like behaviors (impaired reality testing).

## Introduction

Pavlovian conditioning is the process whereby experience with a conditional relationship between a neutral cue [conditioned stimulus (CS)] and an outcome [unconditioned stimulus (US)] enables CS to promote behaviors toward obtaining or avoiding US [conditioned response (CR)]. Since US consists of many different elements, CS may become associated with one or more of these elements (e.g., identity-specific sensory features, general motivational or emotional features, etc.). It has been suggested that the details of US representation accessed by CS determines the behaviors CS can promote (Delamater, 2012) [e.g., different CRs, mediated learning (see below), conditioned reinforcement]. The content of association may, in turn, be determined by a variety of conditioning factors such as interstimulus interval (ISI) and training amount (see below). As an example, eyeblink conditioning experiments on rabbits demonstrate how different CRs can be elicited depending on the ISI parameter (Vandercar and Schneiderman, 1967). After the presentation of a tone CS followed by an eye shock, specific eye blink and heart rate CRs are measured: CS elicits both the CRs when it predicts the eye shock with short latency, but does only heart rate CR when the latency is longer. These results suggest that the specific somatosensory location information is not encoded by CS with a longer ISI. This differential CR is adaptive in that, rabbit protects the specific eye by blinking that eye with a short latency, and it can still run away from the dangerous situation with a longer latency. It is hypothesized that Pavlovian conditioning, through multiple forms of association, allows adaptation to constantly changing environment by shaping behavior according to the current detail of CS-US relationship (Fanselow and Wassum, 2015). The content of association changing with varying conditioning factors, however, has not yet been addressed in relevant brain area signals evoked by CS.

CS comes to activate an internal representation of US after CS-US pairings (McLaren and Mackintosh, 2002; Pearce, 2013), and this associatively-activated representation or “CS-evoked representation” can substitute for the actual US itself in the acquisition of new learning about US, which is called representation-mediated learning (RML) (“mediated learning” in short). Rodents that have received CS-US (tone-sugar or odor-sugar) pairings, later learn to reduce consumption of sugar reward when they are presented with the CS alone paired with lithium chloride (LiCl)-induced nausea (Holland, 1981; Holland and Wheeler, 2009; Kim and Koh, 2016; Busquets-Garcia et al., 2017). The Pairing of CS with a reinforcer can confer a new value to the US that was never directly paired with the reinforcer. The CS serves as a surrogate for its associated sugar stimulus, so that this CS-evoked representation does support taste aversion learning. Mediated learning normally occurs only transiently with “minimal” training in the early stage of the initial conditioning, and CS-nausea pairing can no longer establish an aversion to the US later in the “extended” training stage, so that sensitivity to mediated learning changes over the course of the initial conditioning (Holland, 1998, 2005; Holland and Wheeler, 2009; Kim and Koh, 2016; Busquets-Garcia et al., 2017). Holland et al. hypothesized that with a small number of CS-US pairings CS might evoke a highly realistic representation which is not fully distinguished from the actual US (“conditioned hallucination”) so that mediated learning can occur, and that with extended training CS-evoked representation is replaced by a less perceptual one that is distinguishable from the actual US (“reality testing”), hence mediated learning cannot occur (Holland, 2008; Holland and Wheeler, 2009; McDannald and Schoenbaum, 2009). In other words, the content of association may change over the extended course of training.

Our previous study using an odor-sugar conditioning observed the normal course of mediated learning sensitivity in wild-type (WT) mice by detecting an effect of the amount of initial training (Kim and Koh, 2016). Phospholipase C β1-knockout (PLCβ1-KO) mice, which are one of the genetic mouse models of schizophrenia (Koh et al., 2008; Koh, 2013), display persistent mediated learning. This study aimed to address the hypothetical normal transition in the content of association over the extended course of training, by analyzing patterns of CS-evoked neural activation (c-Fos expression) in the relevant brain regions of PLCβ1-KO and WT mice. The persistent mediated learning in PLCβ1-KO mice, observed in the previous study, suggests that PLCβ1 is necessary for the normal course of mediated learning sensitivity. To study how PLCβ1 is involved in the hypothetical normal transition in the content of association, we first sought in which brain area PLCβ1 is required, by examining the effect of local knockdown on mediated learning sensitivity and CS-evoked neural activation pattern after extended training.

## Materials and Methods

### Subjects

Phospholipase C β1 (PLCβ1) wild-type (WT, *N* = 142) and knockout (KO, *N* = 30) mice (12–15 weeks old) were obtained by mating parental strain C57BL/6J (N30) PLCβ1^+/–^ and 129S4/SvJae (N39) PLCβ1^+/–^ mice. Mice were housed individually in a home cage and deprived of food for 24 h before the start of the experiment. Subjects were maintained on a 12:12 light/dark schedule with a light on at 08:30 AM. All the experiments were performed between 10:00 AM and 4:00 PM and each animal was used only for one experiment. All the experiments including animal care and handling procedures were performed in accordance with the institutional guidelines and regulations of Korea Institute of Science and Technology (KIST). All the experimental protocols were approved by the Institutional Animal Care and Use Committee (IACUC) of KIST (AP201149).

### Odor-Sugar Conditioning and Sampling of Brain Tissue for c-Fos Immunohistochemistry

**FIGURE 1:**
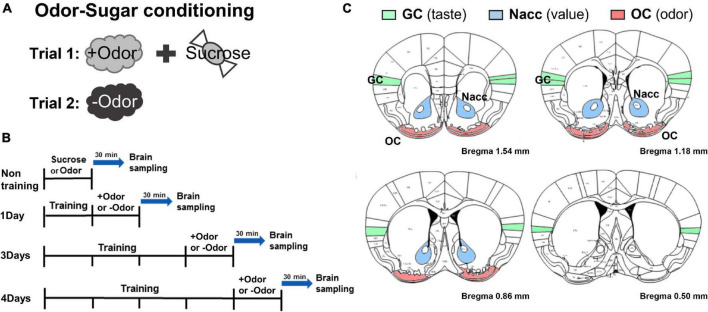
Experimental timeline to examine conditioned stimulus (CS)-evoked neural activation over the 4-day course of odor-sugar conditioning. **(A)** Odor-sugar conditioning. After 2 days of habituation to sugar pellets, a single olfactory training session (trial 1: +Odor → sugar pairing; trial 2: –Odor without sugar) per day was given for various training days. **(B)** Brain sampling. Subjects were divided into non-training, 1Day, 3Days, and 4Days group. The non-training group was exposed to sugar or an odor and 1Day, 3Days, and 4Days group were exposed to +Odor or –Odor on the day after the last training session and then sacrificed 30 min later. **(C)** Schematic representation of the gustatory cortex (GC), the nucleus accumbens (Nacc), and the olfactory cortex (OC) region in mice (bregma 1.54–0.50 mm) (The Mouse Brain in Stereotaxic Coordinates, 2nd edition, by George Paxinos and Keith BJ Franklin).

Odor-sugar Pavlovian conditioning procedure (“olfactory discrimination”) was performed exactly the same as described in our previous study (Kim and Koh, 2016). All the subjects received two trials where one odor (+Odor) is paired with sucrose pellets and the other odor (–Odor) is not paired with sucrose pellets (counterbalanced). These two trials, one for +Odor and another for –Odor, make up one session (Figure 1A). Subjects were trained with one session per day and divided into four groups based on the number of training days: non-training, 1Day, 3Days, and 4Days groups (Figure 1B). 3 days of training (3Days) is defined as “minimal” in that this is the least amount of training for the conditioned response (CR, conditioned approach to the food port, i.e., the relative time spent in food cup in response to +Odor) to reach the plateau in the learning curve, and 4 days (4Days) is defined as “extended,” meaning “more than enough” to reach the plateau (Kim and Koh, 2016). After corresponding days of odor-sugar pairing, 1Day, 3Days, and 4Days groups were exposed to +Odor or –Odor and then sacrificed 30 min later for c-Fos immunohistochemistry. Non-training group was exposed to sucrose or any odor without any prior training and then sacrificed 30 min later.

### Histology for c-Fos Expression in the Gustatory Cortex, Nucleus Accumbens, Olfactory Cortex, and Medial Prefrontal Cortex

Animals were perfused transcardially with saline followed by 10% formalin solution neutral-buffered, under avertin anesthesia [1.5 g/kg intraperitoneal (IP)]. The brains were removed and postfixed in the same fixative for 24 h. Brain tissues were cryoprotected by infiltration with 30% sucrose overnight, frozen, and then sectioned with a cryostat into 40-μm sections, and the consecutive sections were placed in six-well plates containing phosphate-buffered saline (PBS). Sections were floated in 3% hydrogen peroxide (H_2_O_2_) in methanol for 10 min in order to eliminate endogenous peroxidase activity and then in PBS with 0.3% Triton X-100. Thereafter, these sections were incubated with blocking serum in PBS for 1 h and then for 3 days with the antibody to c-Fos protein (Santa Cruz Biotechnology Incorporation, CA, United States; diluted 1:1,000) in blocking serum at 4°C. After being washed in PBS, the sections were incubated in the biotinylated secondary antibody, anti-rabbit immunoglobulin G (IgG) (VECTASTAIN^®^ Elite ABC Kit, CA, United States) for 1 h, followed by incubation in an avidin and biotinylated horseradish peroxidase macromolecular complex (VECTASTAIN^®^ Elite ABC kit, CA, United States) for 1 h. Thereafter, the sections were rinsed with PBS. The sections were then mounted on the subbed slides and coverslipped with mounting medium (VECTOR, VectaMount, CA, United States). We analyzed c-Fos-positive cells in the gustatory cortex (GC), nucleus accumbens (Nacc), olfactory cortex (OC), and medial prefrontal cortex (mPFC) brain areas. The areas were collected by a systematic and random sample of sections according to a brain atlas. Every 6–8th section from bregma 1.98–1.42 mm for the mPFC, 1.42–0.86 mm for the OC, and 1.54–0.74 mm for the GC and Nacc, gave us approximately three random sections for each area per animal. Three sections for each area were photographed by using a microscope equipped with a digital camera (Olympus, Tokyo, Japan). The numbers of c-Fos-positive cells were counted within each brain area by using the Image-J free software (NIH, MD, United States). Bilateral counts per square mm from each section served as a data point. Sections that were damaged or difficult to count c-Fos-positive cells were excluded (Zhang et al., 2006).

### Injection of AAV-shPLCβ1 (or shSCR) -mCherry Virus Into the Medial Prefrontal Cortex

For knockdown of PLCβ1 in the mPFC, we injected the adeno-associated virus (AAV)-shPLCβ1-mCherry virus in WT mice. The AAV-shPLCβ1 virus was created as described previously (Kim et al., 2015). The AAV-shPLCβ1 virus expresses a small hairpin RNA (shRNA) targeting PLC-β1 messenger RNA (mRNA) (target sequence 5′-CCTCCAGTGAGGAGA-TAGAAA-3′). The scrambled shRNA (shSCR) sequence, 5′-AATCG-CATAGCGTATGCCGTT-3′, was used to construct a non-targeting control virus. Both the viruses were created at the KIST Virus Facility. After anesthetizing WT mice with 2% avertin (tribromoethyl alcohol/tertiary amyl alcohol; Aldrich, MA, United States), 0.5 μl of a high-titer AAV preparation (10^13^ GC/ml) was bilaterally (or unilaterally) injected into the mPFC (+1.6 anterior-posterior, ±0.3 mediolateral, and -1.80 dorsoventral) at a rate of 0.05 μl/min by using a Hamilton syringe connected to a microinjection pump (KD Scientific, MA, United States) (Kim et al., 2015). The methods of histology for PLCβ1 expression in the mPFC and quantitative real-time PCR are included in Supplementary Material.

### Representation-Mediated Learning Paradigm

The representation-mediated learning (RML) paradigm consists of an odor-sugar Pavlovian conditioning procedure and a conditioned taste aversion (CTA) paradigm that pairs +Odor with LiCl injection. This procedure is described as “representation-mediated taste aversion” in our previous study (Kim and Koh, 2016). All the subjects (Bi shPLCβ1, *N* = 8; Bi shSCR, *N* = 6; Left shPLCβ1, *N* = 10; Left shSCR, *N* = 11; Right shPLCβ1, *N* = 12; Right shSCR, *N* = 9) were single housed and habituated to pellets by giving them 60 grain-based pellets for 1 h every day for 2 days. The following day, all the subjects were given a single odor-sugar association training session per day, as described in the “odor-sugar conditioning” procedure section above, for 4 days. In the pretest trial of the CTA paradigm, all the subjects were given 60 pellets and the number of pellets consumed in 20 min was counted in a home cage. The next day, all the subjects received LiCl (0.15 M, 30 ml/kg) injection 1.5 min before the presentation of +Odor in the olfactory training box. In the test trial, the number of pellets consumed in 20 min was counted. The aversion index (AI) is [(number of pellets consumed in pretest trial - the number of pellets consumed in test trial)/number of pellets consumed in pretest trial]. Positive numbers indicate that a taste aversion was formed. The following day, CR for the initial olfactory training was measured. The time spent seeking sugar pellets in the food cup in response to each odor during the 90-s period after the odor pumping was measured by analyzing the video records. CR, or the measure of association between +Odor and sugar pellets, is defined as the relative time spent in food cup in response to +Odor: +Odor / total = [time in food cup in response to +Odor / (time in food cup in response to –Odor + time in food cup in response to +Odor)].

### Statistical Analysis

Data were acquired by using the GraphPad Prism 7.03 software (GraphPad, CA, United States). After passing the Shapiro–Wilk normality test, data were analyzed with the ANOVA. *Post hoc* comparisons were made with the Bonferroni’s or Sidak’s multiple comparisons test. A two-group comparison was made by *t*-test. All the data are expressed as mean ± SEM. *p* < 0.05 was considered as statistically significant.

## Results

### Persistent Association With a Sensory Feature of Unconditioned Stimulus in Phospholipase C β1-Knockout Mice

In our previous study (Kim and Koh, 2016) using an representation-mediated learning (RML) procedure that combines a Pavlovian association pairing odor CS with sugar US and a conditioned taste aversion (CTA) method, we compared behaviors of WT and PLCβ1-KO mice. With a “minimal” amount of initial odor-sugar training (one training session a day for 3 days: 3Days), both the WT and PLCβ1-KO mice were able to form an aversion to sugar when the odor CS predicting sugar was paired with LiCl-induced nausea. With an “extended” initial training (one training session a day for 4 days: 4Days), however, only PLCβ-KO mice could form an RML. According to the hypothesis by Holland, it is expected that the nature of the CS-evoked US representation (the content of association) changes from strongly perceptual to less perceptual one over the extended course of training in WT, and that it stays strongly perceptual in PLCβ1-KO mice. This study traced the content of association by assessing CS-evoked neural activation in the gustatory cortex (GC) for the sensory feature of sugar US, the nucleus accumbens (Nacc) for its incentive motivational value (Saunders et al., 2018), and the olfactory cortex (OC) as a control for associative learning (Figure 1C), after minimal or extended training, by using c-Fos immunohistochemistry, in the WT and PLCβ1-KO mice.

#### Patterns of Conditioned Stimulus-Evoked Neural Activation Over the Extended Course of Odor-Sugar Conditioning in Wild-Type and Phospholipase C β1-Knockout Mice

In odor-sugar conditioning, mice are trained to distinguish between the odor paired with sugar pellet reward (+Odor) and the unpaired one (–Odor), so that +Odor (CS) predicts sugar pellets (Figure 1A). The experimental timeline and training procedures are shown in Figure 1B. Subjects were divided into non-training, 1Day, 3Days, and 4Days groups. Before sacrifice, the non-training group was exposed to sugar or an odor only, and 1Day, 3Days, and 4Days groups were exposed to +Odor or –Odor on the day after the last training session. To assess the CS-evoked neural activation pattern, we measured c-Fos immunoreactivity in the GC and the Nacc of the brain samples taken from mice exposed to +Odor relative to –Odor controls.

**FIGURE 2:**
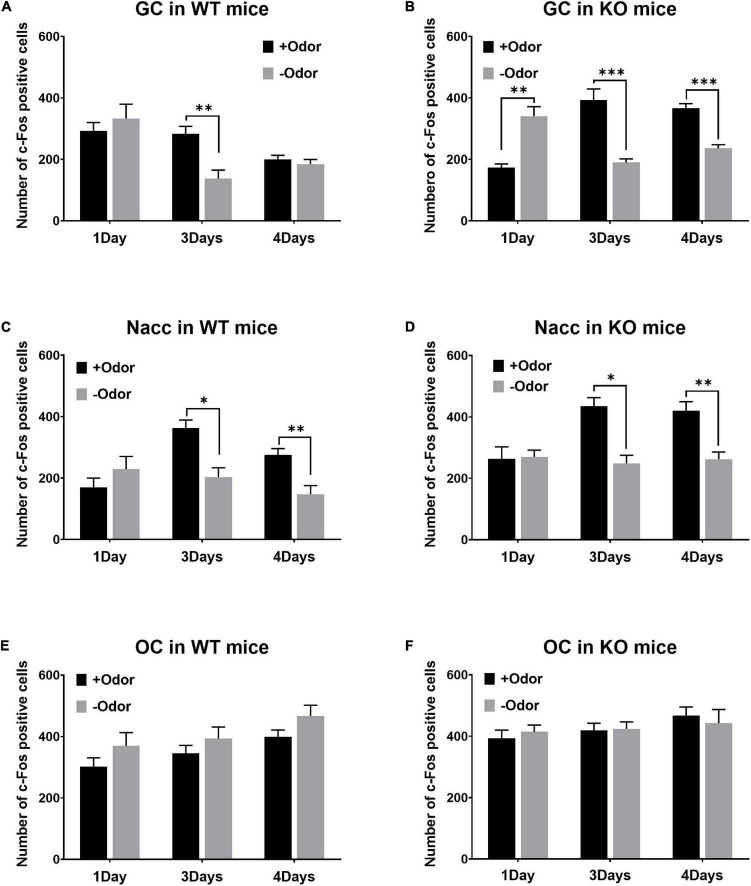
Patterns of CS-evoked neural activation over the 4-day course of odor-sugar conditioning in wild-type (WT) and phospholipase C β1-knockout (PLCβ1-KO) mice. **(A,C,E)** Number of c-Fos-positive cells (per section) after the exposure to +Odor (black) or –Odor (gray), with 1Day, 3Days, or 4Days training condition, in the GC **(A,B)**, the Nacc **(C,D)**, and the OC **(E,F)** of WT and PLCβ1-KO mice. Numbers of mice (*N*) and sections (*n*): **(A)** (WT GC: 1Day +Odor, *N* = 4, *n* = 12; 1Day –Odor, *N* = 3, *n* = 9; 3Days +Odor, *N* = 5, *n* = 15; 3Days –Odor, *N* = 3, *n* = 9; 4Days +Odor, *N* = 10, *n* = 30; 4Days –Odor, *N* = 9, *n* = 27), **(B)** (KO GC: 1Day +Odor, *N* = 3, *n* = 9; 1Day –Odor, *N* = 3, *n* = 9; 3Days +Odor, *N* = 3, *n* = 9; 3Days –Odor, *N* = 3, *n* = 9; 4Days +Odor, *N* = 6, *n* = 18; 4Days –Odor, *N* = 6, *n* = 18), **(C)** (WT Nacc: 1Day +Odor, *N* = 4, *n* = 11; 1Day –Odor, *N* = 3, *n* = 6; 3Days +Odor, *N* = 5, *n* = 15; 3Days –Odor, *N* = 3, *n* = 9; 4Days +Odor, *N* = 10, *n* = 30; 4Days –Odor, *N* = 9, *n* = 18), **(D)** (KO Nacc: 1Day +Odor, *N* = 3, *n* = 9; 1Day –Odor, *N* = 3, *n* = 9; 3Days +Odor, *N* = 3, *n* = 9; 3Days –Odor, *N* = 3, *n* = 9; 4Days +Odor, *N* = 6, *n* = 18; 4Days –Odor, *N* = 6, *n* = 17), **(E)** (WT OC: 1Day +Odor, *N* = 4, *n* = 12; 1Day –Odor, *N* = 3, *n* = 9; 3Days +Odor, *N* = 5, *n* = 15; 3Days –Odor, *N* = 3, *n* = 9; 4Days +Odor, *N* = 10, *n* = 30; 4Days –Odor, *N* = 9, *n* = 22), **(F)** (KO OC: 1Day +Odor, *N* = 3, *n* = 9; 1Day –Odor, *N* = 3, *n* = 9; 3Days +Odor, *N* = 3, *n* = 9; 3Days –Odor, *N* = 3, *n* = 9; 4Days +Odor, *N* = 6, *n* = 18; 4Days –Odor, *N* = 6, *n* = 18). All the values are mean ± SEM. *p < 0.05; ^**^p < 0.01; ^***^p < 0.001.

In the GC of WT mice, +Odor induced significantly higher level of c-Fos expression than –Odor with 3Days condition, but not with 4Days condition (Figure 2A). In the GC of PLCβ1-KO mice, +Odor induced significantly higher c-Fos expression than –Odor with either training condition (Figure 2B). The three-way ANOVA [(genotype— WT, KO) × (training— 1Day, 3Days, 4Days) × (cue— +Odor, –Odor)] for number of c-Fos-positive cells in the GC revealed significant main effects of all the factors [genotype: *F*(1,162) = 11.29, *p* = 0.0010; training: *F*(2,162) = 3.18, *p* = 0.044; cue: *F*(1,162) = 27.46, *p* < 0.0001], no genotype × cue interaction [*F*(1,162) = 0.3065, *p* = 0.58], and significant interactions in other combinations [genotype × training: *F*(2,162) = 14.75, *p* < 0.0001; training × cue: *F*(2,162) = 30.74, *p* < 0.0001; genotype × training × cue: *F*(2,162) = 7.73, *p* = 0.0006]. *Post hoc* comparisons found significant differences in number of c-Fos-positive cells between +Odor and –Odor groups in WT 3Days (*p* = 0.002), KO 1Day (*p* = 0.002), KO 3Days (*p* < 0.0001), and KO 4Days (*p* = 0.0001).

In the Nacc, +Odor induced significantly higher c-Fos expression than –Odor with either training condition in both the genotypes (Figures 2C,D). The three-way ANOVA [(genotype— WT, KO) × (training— 1Day, 3Days, 4Days) × (cue— +Odor, –Odor)] for number of c-Fos-positive cells in the Nacc revealed significant main effects of all the factors [genotype: *F*(1,148) = 21.63, *p* < 0.0001; training: *F*(2,148) = 5.18, *p* = 0.007; cue: *F*(1,148) = 26.60, *p* < 0.0001]. There was a significant training × cue interaction [*F*(2,148) = 10.41, *p* < 0.0001], but no interaction in other combinations [genotype × training: *F*(2,148) = 1.94, *p* = 0.15; genotype × cue: *F*(1,148) = 0.99, *p* = 0.32; genotype × training × cue: *F*(2,148) = 0.05, *p* = 0.9]. *Post hoc* comparisons found significant differences in number of c-Fos-positive cells between +Odor and –Odor groups in WT 3Days (*p* = 0.03), WT 4Days (*p* = 0.005), KO 3Days (*p* = 0.017), and KO 4Days (*p* = 0.001).

There was no significant difference between +Odor and –Odor groups with any training condition in the OC in both the genotypes (Figures 2E,F), excluding the possibility that any significant difference detected in panels A–D was due to difference in olfactory sensory responses to the odors themselves rather than to associative learning. The three-way ANOVA [(genotype— WT, KO) × (training— 1Day, 3Days, 4Days) × (cue— +Odor, –Odor)] for the number of c-Fos-positive cells in the OC revealed significant main effects of genotype [*F*(1,166) = 5.09, *p* = 0.03] and training [*F*(2,166) = 5.14, *p* = 0.006], but no significant effect of cue [*F*(1,166) = 2.14, *p* = 0.15], and no significant interaction (*p* > 0.05) [genotype × training: *F*(2,166) = 0.50, *p* = 0.60; training × cue: *F*(2,166) = 0.10, *p* = 0.90; genotype × cue: *F*(1,166) = 2.085, *p* = 0.90; genotype × training × cue: *F*(2,166) = 0.18, *p* = 0.83].

In all the three brain areas of both the genotypes, there was no significant difference in c-Fos expression between +Odor and –Odor groups with 1Day condition, except for in the GC of PLCβ1-KO mice (Figure 2B) where the c-Fos response to +Odor was significantly lower than that to –Odor. In non-training groups, which were not given odor-sugar conditioning, all the three brain areas appeared to respond appropriately to sugar or odor (Supplementary Figure 1): Sugar, having the taste sensory feature and the appetitive value, activated both the GC and the Nacc significantly more strongly than odor in both the genotypes (Supplementary Figures 1A–D); responses of OC to odor were significantly higher than those to sugar in both the genotypes (Supplementary Figures 1E,F).

In summary, CS-evoked GC activation was present after minimal (3Days) training, and absent after extended (4Days) training in WT mice, but it was present with either training condition in PLCβ1-KO mice. On the contrary, CS-evoked activity in the Nacc was present with either training condition in both the genotypes. Given our previous report (Kim and Koh, 2016) showing that WT mice cannot perform RML after extended training, and that RML can occur with either training condition in PLCβ1-KO mice, this taste sensory encoding concurrent with mediated learning sensitivity suggests that the behavioral difference between these two genotypes comes from the difference in the course of association content. As hypothesized by Holland and his colleagues, the disappearance of sensory association after extended training in WT mice can be a normal training-dependent change in the content of association, the process of which is shown here to be disrupted in PLCβ1-KO mice.

### Mediated Learning Sensitivity and Association With a Sensory Feature of Unconditioned Stimulus in the Left mPFC-Phospholipase C β1-Knockdown Mice That Received Extended Training

The results of CS-evoked neural activation experiments in PLCβ1-KO mice suggest that PLCβ1 is required for the normal training-dependent change in the content of association. PLCβ1 is distributed mainly in the forebrain and the medial prefrontal cortex (mPFC) is one of the areas where it is abundantly expressed (Watanabe et al., 1998; Fukaya et al., 2008; Kim et al., 2015). The mPFC is also shown to have implications for hallucination in recent brain imaging studies. A significant difference in the specific morphological aspect of mPFC has been reported in schizophrenia patients with hallucinations compared to the healthy subjects (Garrison et al., 2015). Functional MRI (fMRI) studies on schizophrenia patients with hallucination suggest that abnormal connectivity between the mPFC and other cortical regions is associated with psychotic conditions (Lawrie et al., 2002; Amad et al., 2014; Scheinost et al., 2018). Given that persistent mediated learning has been proposed as an animal model for hallucination-like behaviors (McDannald and Schoenbaum, 2009; Kim and Koh, 2016; Busquets-Garcia et al., 2017), implicating aberrant associative learning process contributing to hallucinations, these reports suggest the mPFC be first examined in terms of asking how PLCβ1 is involved in this normal Pavlovian association process.

#### Conditioned Stimulus-Evoked Neural Activation in the mPFC Over the Extended Course of Odor-Sugar Conditioning in Wild-Type and Phospholipase C β1-Knockout Mice

**FIGURE 3:**
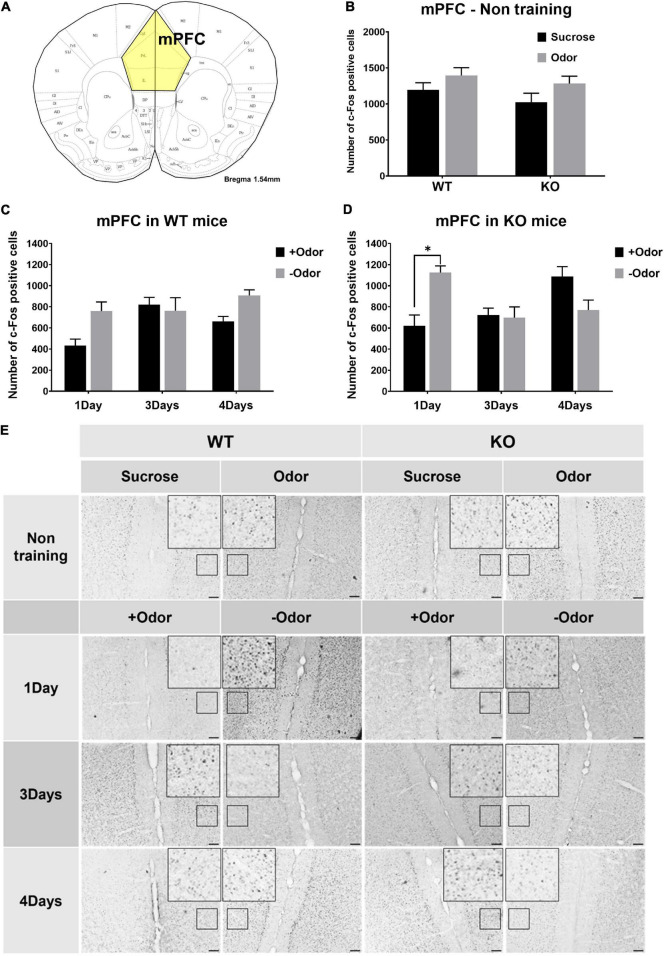
Conditioned stimulus (CS)-evoked neural activation in the mPFC over the course of odor-sugar conditioning in wild-type (WT) and PLCβ1-knockout (KO) mice. **(A)** Schematic representation of the mPFC in mice. **(B)** Number of c-Fos-positive cells in the mPFC after the exposure to sucrose (black) or odor (gray) in non-training group of WT and KO mice. **(C,D)** Number of c-Fos-positive cells in the mPFC after the exposure to +Odor (black) or –Odor (gray) with 1Day, 3Days, or 4Days training condition in WT mice **(C)** and in KO mice **(D)**. **(E)** Representative images of c-Fos immunohistochemistry in the mPFC. Calibration bar is 100 μm. Numbers of mice (*N*) and sections (*n*): **(B)** (mPFC - non training: WT Sucrose, *N* = 4, *n* = 5; WT Odor, *N* = 3, *n* = 6; KO Sucrose, *N* = 3, *n* = 6; KO Odor, *N* = 3, *n* = 6), **(C)** (WT mPFC: 1Day +Odor, *N* = 4, *n* = 8; 1Day –Odor, *N* = 3, *n* = 6; 3Days +Odor, *N* = 5, *n* = 10; 3Days –Odor, *N* = 3, *n* = 6; 4Days +Odor, *N* = 10, *n* = 14; 4Days –Odor, *N* = 9, *n* = 18), **(D)** (KO mPFC: 1Day +Odor, *N* = 3, *n* = 6; 1Day –Odor, *N* = 3, *n* = 6; 3Days +Odor, *N* = 3, *n* = 6; 3Days –Odor, *N* = 3, *n* = 6; 4Days +Odor, *N* = 6, *n* = 11; 4Days –Odor, *N* = 6, *n* = 9). All the values are mean ± SEM. *p < 0.05.

In the experiments examining neural activation in response to odor CS, an additional analysis was done for the mPFC to examine whether the lack of PLCβ1 would cause any difference in the responses of the mPFC to CS over the extended course of odor-sugar conditioning (Figure 3). In non-training condition, sucrose- or odor-evoked mPFC activities were not different among the groups (Figure 3B), indicating similar levels of general activation in response to odor and taste stimuli in the two genotypes. The one-way ANOVA [(genotype— WT, KO) × (stimulus— Sucrose, Odor)] for the number of c-Fos-positive cells in the mPFC revealed no significant main effect or interaction (*p* > 0.05) [genotype: *F*(1,19) = 1.62, *p* = 0.21; stimulus: *F*(1,19) = 0.07, *p* = 0.79; interaction: *F*(1,19) = 0.07, *p* = 0.8].

Not much difference between the two genotypes was observed in the responses of the mPFC to CS over the extended course of odor-sugar conditioning, except that, with one-day training condition (1Day), KO mice showed a significantly higher mPFC response to –Odor than to +Odor (Figures 3C,D). The three-way ANOVA [(genotype— WT, KO) × (training— 1Day, 3Days, 4Days) × (cue— +Odor, –Odor)] for the number of c-Fos-positive cells in the mPFC revealed significant main effects of all the factors [genotype: *F*(1,92) = 5.63, *p* = 0.02; training: *F*(2,92) = 3.12, *p* = 0.049; cue: *F*(1,92) = 5.58, *p* = 0.02], no significant genotype × cue interaction [*F*(1,92) = 1.53, *p* = 0.22], and significant interactions in other combinations [genotype × training: *F*(2,92) = 4.13, *p* = 0.019; training × cue: *F*(2,92) = 9.29, *p* = 0.0002; genotype × training × cue: *F*(2,143) = 6.72, *p* = 0.001]. *Post hoc* comparisons found a significant difference in number of c-Fos-positive cells between +Odor and –Odor groups in KO 1Day (*p* = 0.016).

#### Effects of Phospholipase C β1 Knockdown in the Bilateral, Left, and Right mPFC on Mediated Learning Sensitivity After Extended Training

Studies with schizophrenia patients suggest that left frontotemporal dysfunction or reduced connectivity is associated with sensory hallucinations (Lavigne et al., 2015; Kobayashi, 2018). In addition, an electroencephalography (EEG) study on PLCβ1-KO mice showed that the left frontal-auditory cortex connectivity is significantly reduced compared to WT mice (Shahriari et al., 2016). These reports suggest that there may be functional lateralization of the frontal cortical areas involved in the hallucination-like behaviors. We, thus, sought to examine the effects of local knockdown of PLCβ1 in the bilateral, left, or right mPFC by injecting shPLCβ1 or shSCR virus (Supplementary Figure 2). In order to study the role of PLCβ1 in the normal training-dependent transition in the content of association, we first examined the effects of local knockdown on mediated learning sensitivity after extended training, at which point the content of association changes, using an RML procedure as described in our previous study (Kim and Koh, 2016).

**FIGURE 4:**
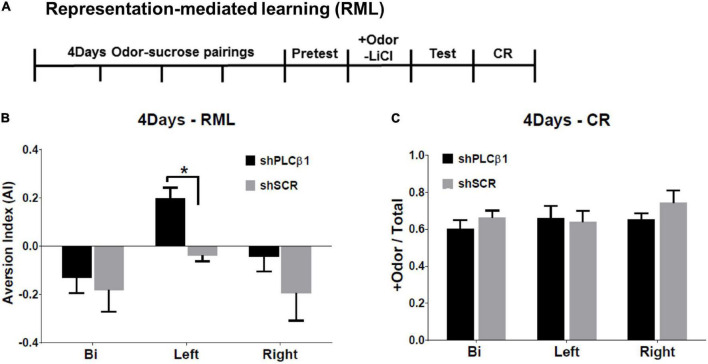
Effects of local knockdown of Phospholipase C β1 (PLCβ1) in the bilateral, left, or right medial prefrontal cortex (mPFC) on mediated learning sensitivity after extended training (4Days). **(A)** Representation-mediated learning (RML) paradigm took place over 8 days. +Odor → sugar conditioning was given for 4 days. On day 5, a baseline sugar consumption was measured. On day 6, +Odor → nausea pairing was given. A post-nausea sugar consumption was measured on day 7 and conditioned response (CR) was tested on day 8. **(B)** Aversion index (AI) values for the mice injected, bilaterally (Bi), to the left (Left), or right (Right) side of mPFC, with shPLCβ1 (black) or shSCR (gray) virus. **(C)** CR, the relative time spent in food cup in response to +Odor, tested after RML test for the same groups shown in panel **(B)**. Numbers of mice (*N*): **(B)** (Bi shPLCβ1, *N* = 8; Bi shSCR, *N* = 6; Left shPLCβ1, *N* = 10; Left shSCR, *N* = 12; Right shPLCβ1, *N* = 12; Right shSCR, *N* = 9), **(C)** (Bi shPLCβ1, *N* = 8; Bi shSCR, *N* = 6; Left shPLCβ1, *N* = 8; Left shSCR, *N* = 3; Right shPLCβ1, *N* = 5; Right shSCR, *N* = 3). All the values are mean ± SEM. *p < 0.05.

The RML procedure combines an odor-sugar conditioning and a conditioned taste aversion method. The experimental timeline is shown in Figure 4A. The shPLCβ1- or shSCR-injected mice were trained for 4 days for olfactory discrimination. After the +Odor → sugar conditioning, a baseline sugar consumption was observed (Pretest). The next day, all the subjects received a pairing of +Odor and LiCl-induced nausea (+Odor → nausea) without sugar pellets present, then a post-nausea sugar consumption was measured the following day (Test). Associative learning by the initial odor-sugar conditioning was examined on the next day with CR, which is measured as the relative time spent seeking reward in the food cup in response to +Odor.

Only the left shPLCβ1-injected mice formed a significant aversion to sugar pellets (Figure 4B). With extended initial training, the left-mPFC-PLCβ1-KD, but neither the bilateral- nor the right-mPFC-KD (knockdown), mice established a mediated learning. The one-way ANOVA [(region— Bi, Left, Right) × (virus— shPLCβ1, shSCR)] for AI found main effects of region [*F*(2,50) = 7.091, *p* = 0.0019] and virus [*F*(1,50) = 6.601, *p* = 0.013], but no interaction [*F*(2,50) = 0.82, *p* = 0.44]. *Post hoc* comparisons found a significant difference in AI between left shPLCβ1- and left shSCR-injected mice (*p* = 0.04). There was no significant difference in CR among groups (Figure 4C) showing that the inability to establish mediated learning was not due to a loss of the association between + Odor and sugar reward. The one-way ANOVA [(region— Bi, Left, Right) × (virus— shPLCβ1, shSCR)] for CR revealed no significant main effect or interaction (*p* > 0.05) [region: *F*(2,27) = 2.72, *p* = 0.08; virus: *F*(1,27) = 2.37, *p* = 0.14; interaction: *F*(2,27) = 1.12, *p* = 0.34].

#### Effect of Phospholipase C β1 Knockdown in the Left mPFC on the Pattern of Conditioned Stimulus-Evoked Neural Activation After Extended Training

As shown in our previous study and Figure 2, WT mice normally lose mediated learning sensitivity and CS-evoked GC activation, but PLCβ1-KO mice display mediated learning and CS-evoked GC activation after extended training. Since PLCβ1 knockdown in the left mPFC enables mediated learning after extended training (Figure 4B), it is expected for CS-evoked GC activation to be detected in the left-mPFC-PLCβ1 knockdown mice that received extended training. To further examine the suggested link between the sensory encoding and mediated learning sensitivity, we, therefore, examined the effect of PLCβ1 knockdown in the left mPFC on the content of association by measuring CS-evoked neural activation in the three brain regions (GC, NAcc, and OC).

**FIGURE 5:**
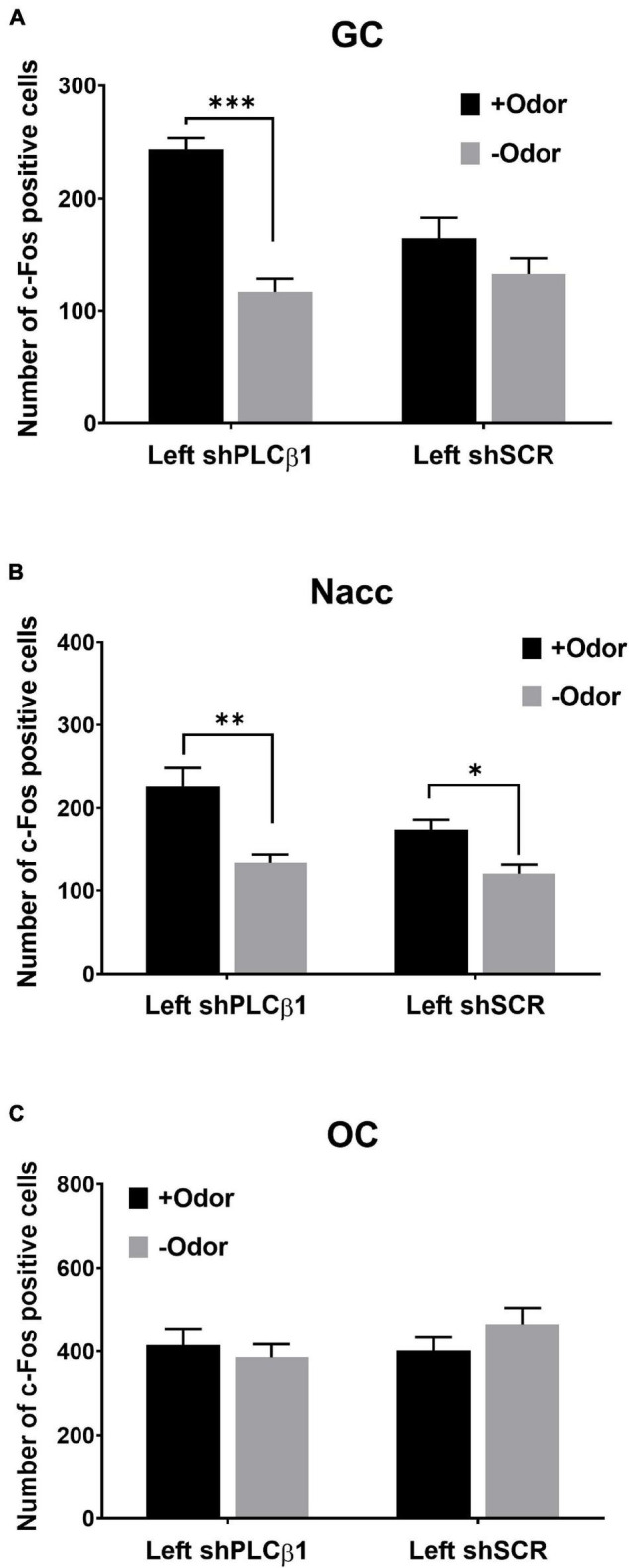
Patterns of CS-evoked neural activation in the left mPFC shPLCβ1- or shSCR-injected mice that received extended training (4Days). Number of c-Fos-positive cells after the exposure to +Odor (black) or –Odor (gray) in the GC **(A)**, the Nacc **(B)**, and the OC **(C)** of left shPLCβ1- or left shSCR-injected mice that received extended training. Numbers of mice (*N*) and sections (*n*): **(A)** (shPLCβ1 +Odor, *N* = 3, *n* = 9; shPLCβ1 –Odor, *N* = 3, *n* = 9; shSCR +Odor, *N* = 3, *n* = 9; shSCR –Odor, *N* = 3, *n* = 9), **(B)** (shPLCβ1 +Odor, *N* = 3, *n* = 9; shPLCβ1 –Odor, *N* = 3, *n* = 9; shSCR +Odor, *N* = 3, *n* = 9; shSCR –Odor, *N* = 3, *n* = 9), **(C)** (shPLCβ1 +Odor, *N* = 3, *n* = 9; shPLCβ1 –Odor, *N* = 3, *n* = 9; shSCR +Odor, *N* = 3, *n* = 9; shSCR –Odor, *N* = 3, *n* = 9). All the values are mean ± SEM. *p < 0.05; ^**^p < 0.01; ***p < 0.001.

In the GC of the left shPLCβ1-injected mice, +Odor induced a significantly higher level of c-Fos expression than –Odor, but not in the left shSCR-injected mice (Figure 5A). The two-way ANOVA [(virus— shPLCβ1, shSCR) × (cue— +Odor, –Odor)] for the number of c-Fos-positive cells in the GC revealed significant main effects and interaction [virus: *F*(1,32) = 4.99, *p* = 0.03; cue: *F*(1,32) = 11.19, *p* = 0.002; interaction: *F*(1,32) = 30.68, *p* < 0.0001]. *Post hoc* comparisons found a significant difference in number of c-Fos-positive cells between +Odor and –Odor groups in the shPLCβ1-injected mice (*p* < 0.0001).

In the Nacc, +Odor induced significantly higher c-Fos expression than –Odor in both the left shPLCβ1- and the left shSCR-injected mice (Figure 5B). The two-way ANOVA [(virus— shPLCβ1, shSCR) × (cue— +Odor, –Odor)] for the number of c-Fos-positive cells in the Nacc revealed significant main effects of both the factors [virus: *F*(1,32) = 4.83, *p* = 0.03; Cue: *F*(1,32) = 24.67, *p* < 0.0001], but no interaction [*F*(1,32) = 1.75, *p* = 0.2]. *Post hoc* comparisons found a significant difference in number of c-Fos-positive cells between +Odor and –Odor groups in both the left shPLCβ1- (*p* = 0.0002) and left shSCR- (*p* = 0.029) injected mice. In the OC, +Odor- and –Odor-evoked activities were not different in both the mice (Figure 5C). The two-way ANOVA [(virus— shPLCβ1, shSCR) × (cue— +Odor, –Odor)] for the number of c-Fos-positive cells in the OC revealed no significant main effect or interaction (*p* > 0.05) [virus: *F*(1,32) = 0.85, *p* = 0.36; cue: *F*(1,32) = 0.24, *p* = 0.63; interaction: *F*(1,32) = 1.71, *p* = 0.2].

These results show that odor CS can evoke the taste sensory representation of sugar US in the left-mPFC-PLCβ1-KD mice that received extended training. Injection of shPLCβ1 virus in the bilateral- or right mPFC did not affect CS-evoked neural activation pattern after extended training (Supplementary Figure 3), which is as expected from the absence of its effect on mediated learning sensitivity (Figure 4B).

## Discussion

This study is the first to show the training amount-dependent transition in the content of association in appetitive Pavlovian conditioning and to propose a molecular component of the neural system underlying this transition. WT mice, which lose mediated learning sensitivity after extended training, also lost the sensory component of CS-evoked US representation after extended training. PLCβ1-KO mice, which display persistent mediated learning, also showed persistent association of CS with the sensory feature of sugar US. These results demonstrate that: (i) stronger sensory nature of CS-evoked US representation (i.e., sensory element included in the content of association) is predictive of mediated learning sensitivity; (ii) training-dependent transition in the content of association from “highly perceptual” to “less perceptual” determines the normal transition from mediated learning sensitivity to reality testing; and (iii) PLCβ1 is required for this transition. This is the first example substantiating the general hypothesis that Pavlovian conditioning allows animal to shape its behavior to environment by forming different forms of associations according to the current nature of CS-US relationship (e.g., how many paired events it has gone through). PLCβ1 knockdown in the left mPFC enabled mediated learning and CS-evoked neural activation in the GC after extended training. No effect of both the right- and bilateral knockdown suggests that there are interactions between the right and left mPFC PLCβ1 signaling systems, and that lower PLCβ1 expression in the left than the right side might lead to an aberrant effect. These results show that balanced expression and signaling of PLCβ1 in the left mPFC is required for this training-dependent change in the content of association.

A recent series of studies using a sensory preconditioning paradigm (Busquets-Garcia et al., 2017, 2018) showed that hippocampal type 1 cannabinoid receptor (CB1R) expression increased by minimal preconditioning training (three odor-taste pairings) leads to synaptic plasticity, and that it controls “incidental association” between low-salience sensory cues, which in turn enables mediated learning. Here, extended training (six pairings) also suppresses mediated learning, and the once-increased CB1R expression during minimal training becomes normalized by extended training. These findings raise a possibility that similar or even the same molecular/synaptic events might be recruited while the very sensory association is formed by minimal training in appetitive Pavlovian conditioning. Future studies need to examine whether similar molecular/cellular changes (e.g., CB1R expression) happen in consistence with the training-dependent change in the content of association in our odor-sugar conditioning paradigm. Conversely, it will be worthwhile to examine whether there is a training-dependent change in odor-evoked neural activation (i.e., GC) pattern in the odor-taste sensory preconditioning paradigm. If it was the case, this study would implicate similar top-down influences on higher-order conditionings, such as sensory preconditioning. Then, it should be examined whether PLCβ1 knockdown or knockout in the left mPFC may also enable mediated learning or odor-evoked GC activation and cause an increased dorsal hippocampal CB1R expression after extended training (six odor-taste pairings). In the same vein, any left mPFC PLCβ1 neurons projecting to the dorsal hippocampus should be explored by using a retrograde tracing method combined with PLCβ1 immunolabeling. This approach might help to investigate putative common mechanisms for the training-dependent top-down control over the sensory stimulus-stimulus associations in both the appetitive Pavlovian conditioning and sensory preconditioning.

Mediated learning, higher-order learning, more generally, is adaptive in that it is likely to increase the chance of survival by accordingly modified responses to relevant events (Gewirtz and Davis, 2000). Depending on the ethological and environmental context (e.g., how the experiences of the associated events are temporally distributed), however, sensory/incidental association and subsequent mediated learning are not always beneficial and could be even disadvantageous, especially when animals need to process the associated information only in the abstract terms. If set out of adaptive context, it may also contribute to psychiatric conditions, such as posttraumatic stress disorder (PTSD) (Wessa and Flor, 2007) and hallucination-like states. Adult rats given neonatal ventral hippocampal lesions, a neurodevelopmental model of schizophrenia, displayed an overall “enhanced” RML after light → sugar initial conditioning (McDannald et al., 2011). Not long after our report on the “persistent” RML observed in PLCβ1-KO mice with odor → sugar initial conditioning (Kim and Koh, 2016), Busquets-Garcia et al. (2017) also found that a similar “persistent” RML can happen in psychotogenic drug-induced (e.g., Δ^9^-tetrahydrocannabinol, MK-801, amphetamine) mouse models of psychotic-like states in a different odor → sugar conditioning paradigm. Another recent study on a mouse model of schizophrenia reported that mice exposed to ketamine during late adolescence subsequently showed a “greater tendency” for RML compared to control mice, with yet another odor → sugar conditioning method (Koh et al., 2018). These studies using various models, conditioning methods, and sensory modalities, present a quasi-universal feature of RML linked to psychotic-like states (impaired reality testing), whether it is about persistence in or overall propensity for RML. Depending on the type of conditioning paradigm, especially when a large number of pairings are required to reach the plateau in learning (e.g., tone or light CS), individual variation might make it difficult to define minimal and extended training conditions in control animals. This technical problem leads to the observation of overall enhanced RML or greater tendency for RML in some models, rather than a disruption of the normal transition in RML sensitivity.

A recent study involving behavioral experiments carried out on both healthy- and psychiatrically-diagnosed human subjects reported that after receiving prior visual → auditory conditioning (checkerboard → tone) daily-hallucinating humans showed a greater tendency for conditioned hallucination (e.g., hearing a tone in response to exposure to checkerboard alone) compared to non-hallucinators (Powers et al., 2017; Dwyer, 2018). Besides, the accompanied functional imaging analysis suggested that the brain activity during conditioned hallucination overlapped with tone-responsive brain regions including the auditory cortex. Here, again, a more detailed assessment regarding training-dependent change needs to be addressed. Nevertheless, these findings from human and animal studies suggest that disruption of training-dependent change or heightened sensory encoding in associative learning is correlated with a propensity for hallucinations or psychotic-like states, validating the merit of the Pavlovian association paradigm as a tool for translational approach.

Phospholipase C β1 is linked to G_q/11_-coupled receptors, among which are muscarinic receptors M1, M3, and M5 (Gutkind et al., 1991). Blockade of muscarinic receptors is known to induce hallucinations (Perry and Perry, 1995) and scopolamine (a competitive muscarinic antagonist) increased the tendency for conditioned hallucination in human subjects who received prior light → tone pairings (Warburton et al., 1985). The main acetylcholine release in the mPFC is from the nucleus basalis of Meynert (NBM). Therefore, it will be interesting to examine a possible involvement of NBM in the training-dependent change in the association content. The mPFC has mainly M1 in both excitatory and inhibitory neurons (Oda et al., 2018). M1 agonist xanomeline is known to significantly relieve hallucinations in patients with Alzheimer’s disease (Bodick et al., 1997). Taken together, we may seek, for instance, circuits or neural systems that are controlled by cholinergic input through M1 receptors in the left mPFC.

It will be intriguing to examine whether this training-dependent change as shown in CS-evoked neural activation pattern and its disruption can be observed also in other rodent models (even other vertebrate species) with a variety of conditioning paradigms of various sensory modalities. To confirm the causal relationship between the sensory encoding by CS and mediated learning sensitivity, our future study will examine the correlation between them. We will also test whether, in WT mice with minimal initial training (3Days), mediated learning can be suppressed by optogenetically inhibiting the GC neurons during the presentation of +Odor in the +Odor → nausea pairing session. Studying physiology of the conditioning factor-dependent change in the content of association, using this model with a clearly defined training amount factor, will not only allow us to ask new important questions about Pavlovian association itself but also help to open new avenues to study the neurobiology of hallucination-like behaviors.

## Data Availability Statement

The raw data supporting the conclusions of this article will be made available by the authors, without undue reservation.

## Ethics Statement

The animal study was reviewed and approved by the institutional Animal Care and Use Committee (IACUC) of KIST (AP201149).

## Author Contributions

H-YK designed the study, interpreted the results, and wrote the manuscript. H-jK set up and performed the experiments and analysis of data. Both authors discussed, contributed to the article, and approved the submitted version.

## Conflict of Interest

The authors declare that the research was conducted in the absence of any commercial or financial relationships that could be construed as a potential conflict of interest.

## Publisher’s Note

All claims expressed in this article are solely those of the authors and do not necessarily represent those of their affiliated organizations, or those of the publisher, the editors and the reviewers. Any product that may be evaluated in this article, or claim that may be made by its manufacturer, is not guaranteed or endorsed by the publisher.
