# Supplementary material for: Training-Dependent Change in Content of Association in Appetitive Pavlovian Conditioning
Source: Front Behav Neurosci. 2021 Nov 25;15:750131. doi: 10.3389/fnbeh.2021.750131 (PMC8656303; doi:10.3389/fnbeh.2021.750131)
Supplement: Supplementary file 1 [file Image_1.pdf]

Supplementary Material

1. Supplementary Methods

1.1. Histology for PLCβ1 expression in the mPFC

Six consecutive sections in the series throughout the entire mPFC from the virus-injected mice were used for immunohistochemistry. Sections were stained by incubation with rabbit anti-PLCβ1 primary antibody (1:100; Santa Cruz Biotechnology) and anti-rabbit-488 conjugated secondary antibody (1:200; Sigma). Sections were mounted in Vectashield mounting media with diamidino-2-phenylindole (DAPI; Vector Laboratories). Thereafter, images were captured and analyzed using an Olympus FluoView FV1000 Confocal Microscope System. For quantification of PLCβ1 immunofluorescence in RNA interference (RNAi)-mediated PLCβ1 knockdown mice, we have performed the cell count in the mPFC according to the previous description (Kim, Seo et al. 2015). Briefly, PLCβ1 immunofluorescent images were captured in the same regions (400 × 400 μm) throughout the sections. Thereafter, the number of PLCβ1-positive cells was counted within these images. All immunoreactive cells were counted regardless of their fluorescence intensities. The percentage of PLCβ1 positive cells in the mPFC of virus-injected mice was estimated as follows: [(the number of PLCβ1 positive cells among the viral vector-infected cells) / (the number of the viral vector-infected cells in the mPFC)] X 100.

1.2. Quantitative real-time PCR

Total cellular RNA was isolated from brain sample using the total RNA extraction kit (Geneall, Korea). cDNA was synthesized using the SensiFAST cDNA synthesis Kit (Bioline). Quantitative real-time PCR (qRT-PCR) was done using a real-time PCR system (Applied Bioline: 1 cycle at 50°C for 2 min & 95°C for 10 min; 40 cycles at 95°C for 15 s & 60°C for 1 min). Relative quantification of cDNA was performed with LightCycler 480 SW 1.5 software (Roche), using the comparative cycle threshold (C_T) method, wherein target C_T levels are normalized to Gapdh C_T values from the same sample (separate wells). The normalized C_T values are then compared with a calibrator value to determine the relative fold changes in target mRNA levels over the calibrator Taqman Probes (Applied Bioline). 20-μl reaction solution (in distilled water) consists of 2.5 μl cDNA, 10 μl Taqman Universal Gene Expression Master Mix (Applied Bioline), and 2 μl of a specific Taqman Probe. Taqman Probes are used to detect the expression of mouse PLCβ1 or housekeeping gene Gapdh (Assay ID; Mm.PT.56a.12599823 or Mm.PT.39a.1) for normalization of total cDNA/sample.

2. Supplementary Figures

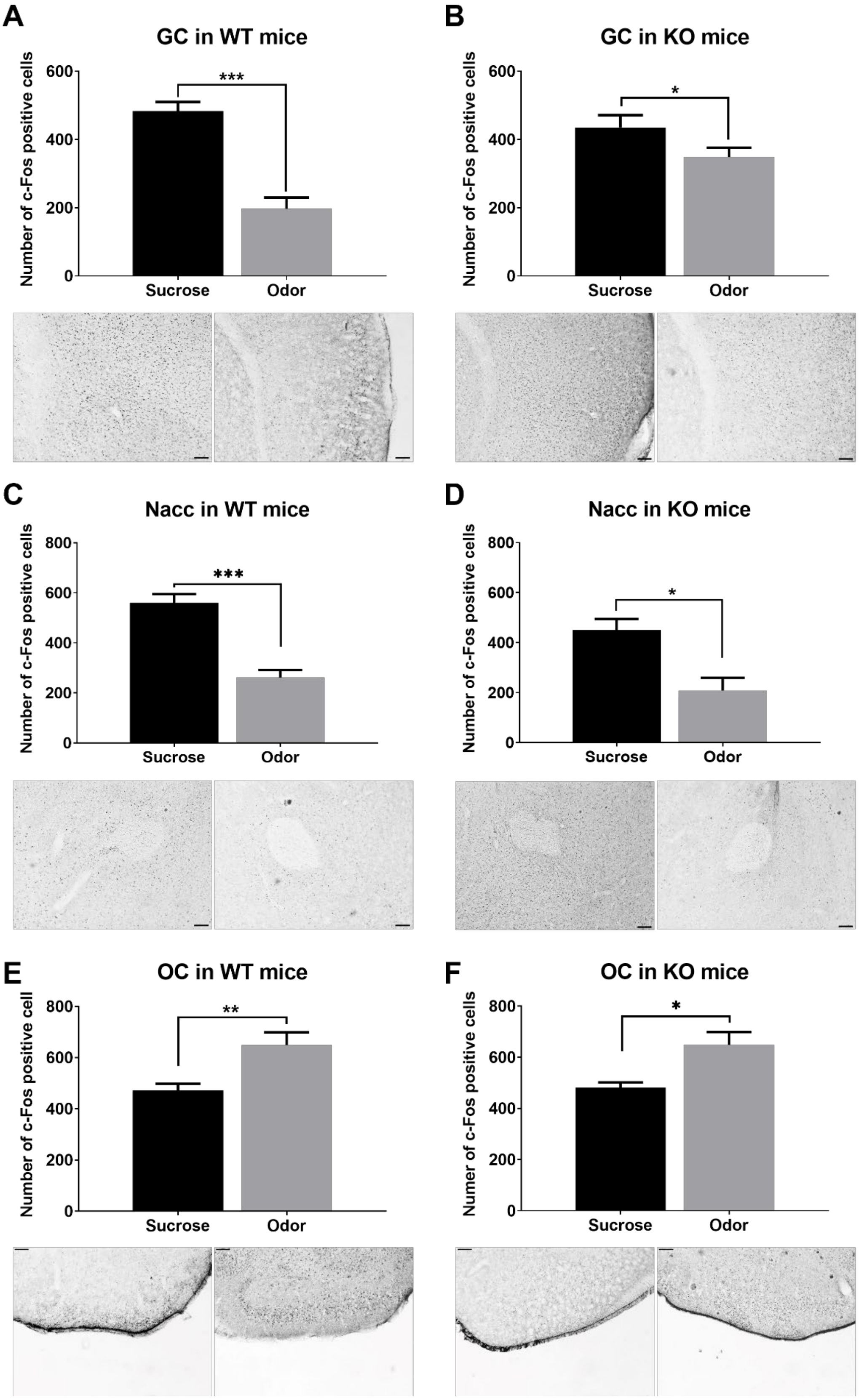

Supplementary Figure 1. Odor- or sucrose- evoked c-Fos expression in the GC, Nacc, and OC of WT and PLCβ1-KO mice with non-training condition. (A, B) Number of c-Fos positive cells after the exposure to Sucrose (black) or Odor (gray) in the GC of WT **(A)** and KO mice **(B)**. Two way-ANOVA [(Genotype- WT, KO) X (Stimulus- Sucrose, Odor)] for number of c-Fos positive cells in the GC revealed a significant main effect of stimulus [$F(1,35) = 43.63$, $p < .001$], but no genotype effect [$F(1,35) = 1.08$, $p = .31$], and a significant interaction [$F(1,35) = 6.69$, $p = .014$]. Post hoc comparisons found a significant difference in number of c-Fos positive cells between Sucrose and Odor groups in both WT ($p < .0001$) and KO ($p = .05$) mice. (WT Sucrose, N = 4, n = 12; WT Odor, N = 3, n = 9; KO Sucrose, N = 3, n = 9; KO Odor, N = 3, n = 9). **(C, D)** Number of c-Fos positive cells in the Nacc of WT **(C)** and KO mice **(D)**. Two way-ANOVA [(Genotype- WT, KO) X (Stimulus- Sucrose, Odor)] for number of c-Fos positive cells revealed a significant main effect of stimulus [$F(1,31) = 44.12$, $p < .001$], but no effect of nor any interaction with genotype (*p*s > .05). Post hoc comparisons found a significant difference in number of c-Fos positive cells between Sucrose and Odor groups in both WT ($p < .0001$) and KO ($p = .0031$) mice (WT Sucrose, N = 4, n=12; WT Odor, N = 3, n = 9; KO Sucrose, N = 3, n = 8; KO Odor, N = 3, n = 6). **(E, F)** Number of c-Fos positive cells in the OC of WT **(E)** and KO mice **(F).** Two way-ANOVA [(Genotype- WT, KO) X (Stimulus- Sucrose, Odor)] for number of c-Fos positive cells in the OC revealed a significant main effect of stimulus [$F(1,33) = 19.81$, $p < .0001$], but no effect of nor any interaction with genotype (*p*s > .89). Post hoc comparisons found a significant difference in number of c-Fos positive cells between Sucrose and Odor groups in both WT ($p = .014$) and KO ($p = .03$) mice (WT Sucrose, N = 4, n = 10; WT Odor, N = 3, n = 9; KO Sucrose, N = 3, n = 9; KO Odor, N = 3, n = 9). Representative image of c-Fos-immunohistochemistry is shown under each bar graph. These patterns are what could be expected from the normal sensory responses of naïve animals. Calibration bar is 100 μm. All values are Mean ± SEM. *$p < .05$; **$p < .01$; ***$p < .001$.

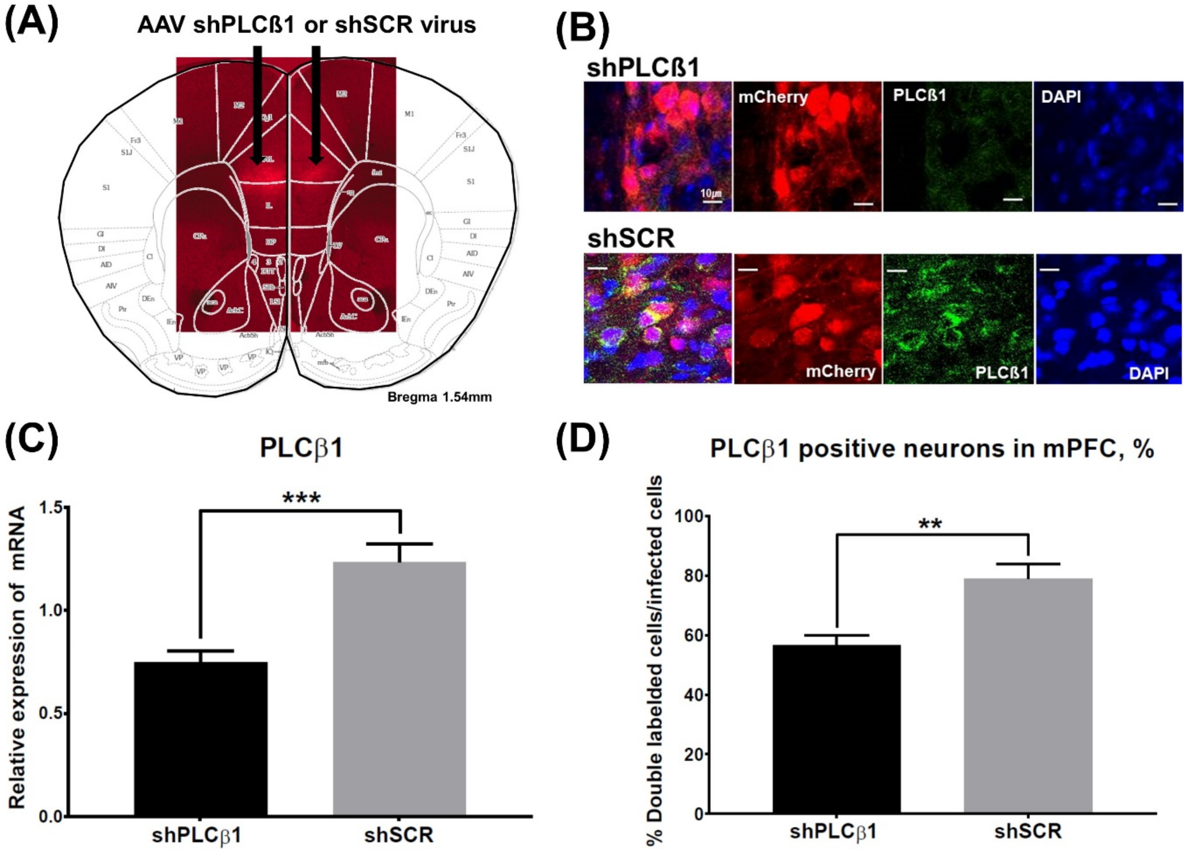

Supplementary Figure 2. PLCβ1 knockdown in the mPFC by injecting mCherry-small hairpin RNA (shRNA) Adeno-associated virus. (A) Adeno-associated virus vectors expressing shRNA for PLCβ1 (shPLCβ1) were bilaterally injected into the mPFC of a mouse brain. mCherry expression under the control of the synapsin-1 promoter was detected in the mPFC. **(B)** Immunofluorescence staining for PLCβ1 expression in the mPFC of mice injected with shPLCβ1 and shSCR. **(C)** Relative expression of PLCβ1 mRNA in fold change is significantly lower in shPLCβ1- than shSCR-injected mice ($p < 0.001$, unpaired t-test) (shPLCβ1, N = 4, n = 25; shSCR, N = 5, n = 8). **(D)** Proportion of PLCβ1 positive neurons among virus-infected ones in the mPFC is significantly lower in shPLCβ1- than shSCR-injected mice ($p = 0.0015$, unpaired t-test) (shPLCβ1, N = 6, n = 8; shSCR, N = 6, n = 11). All values are Mean ± SEM. **$p < 0.01$, ***$p < 0.001$.

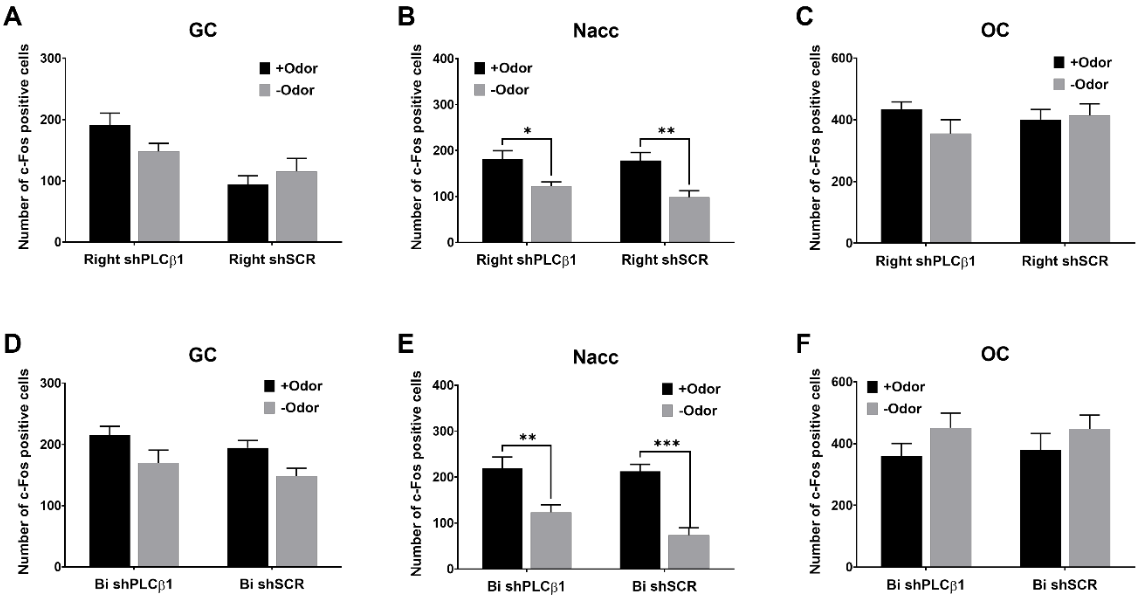

Supplementary Figure 3. Patterns of CS-evoked neural activation in the right, or bilateral mPFC virus-injected mice that received extended training. (A, B, C) Number of c-Fos positive cells after the exposure to +Odor (black) or -Odor (gray) in the GC **(A)**, Nacc **(B)**, and OC **(C)** of the Right shPLCβ1- or shSCR-injected mice that received extended training. **(A)** Two way-ANOVA [(Virus- Right shPLCβ1, Right shSCR) X (Cue– +Odor, -Odor)] for number of c-Fos positive cells in the GC revealed no significant main effect or interaction (*p*s > .05) (Right shPLCβ1 +Odor, N = 3, n = 6; Right shPLCβ1 -Odor, N = 3, n = 6; Right shSCR +Odor, N = 3, n = 5; Right shSCR -Odor, N = 2, n = 5). **(B)** Two way-ANOVA [(Virus- Right shPLCβ1, Right shSCR) X (Cue– +Odor, -Odor)] for number of c-Fos positive cells in the Nacc revealed a significant main effect of virus [$F(1,17) = 19.43$, $p = .0004$], but no effect of nor any interaction with cue (*p*s > .40). Post hoc comparisons found a significant difference in number of c-Fos positive cells between +Odor and –Odor groups in both the right shPLCβ1- ($p = .02$) and right shSCR- ($p = .007$) injected mice (Right shPLCβ1 +Odor, N = 3, n = 6; Right shPLCβ1 -Odor, N = 3, n = 6; Right shSCR +Odor, N = 3, n = 6; Right shSCR -Odor, N = 2, n = 4). **(C)** Two way-ANOVA [(Virus- Right shPLCβ1, Right shSCR) X (Cue– +Odor, -Odor)] for number of c-Fos positive cells in the OC revealed no significant main effect or interaction (*p*s > .1) (Right shPLCβ1 +Odor, N = 3, n = 9; Right shPLCβ1 -Odor, N = 3, n = 6; Right shSCR +Odor, N = 3, n = 9; right shSCR -Odor, N = 2, n = 6). **(D, E, F)** Number of c-Fos positive cells in the GC **(D)**, Nacc **(E)**, and OC **(F)** of Bi shPLCβ1- or shSCR-injected mice. **(D)** Two way-ANOVA [(Virus- Bi shPLCβ1, Bi shSCR) X (Cue– +Odor, -Odor)] for number of c-Fos positive cells in the GC revealed a significant main effect of cue [$F(1,18) = 8.23$, $p = .01$] but no effect of nor any interaction with virus (*p*s > .1) (Bi shPLCβ1 +Odor, N = 3, n = 5; Bi shPLCβ1 -Odor, N = 3, n = 6; Bi shSCR +Odor, N = 3, n = 5; Bi shSCR -Odor, N = 3, n = 6). **(E)** Two way-ANOVA [(Virus- Bi shPLCβ1, Bi shSCR) X (Cue– +Odor, -Odor)] for number of c-Fos positive cells in the Nacc revealed a significant main effect of cue [$F(1,18) = 45.31$, $p < .0001$], but no effect of nor any interaction with virus (*p*s > .1). Post hoc comparisons found a significant difference in number of c-Fos positive cells between +Odor and –Odor groups in both Bi shPLCβ1- ($p = .005$) and Bi shSCR- ($p = .0001$) injected mice (Bi shPLCβ1

+Odor, N = 3, n = 4; Bi shPLCβ1 -Odor, N = 3, n = 6; Bi shSCR +Odor, N = 3, n = 6; Bi shSCR -Odor, N = 3, n = 6). **(F)** Two way-ANOVA [(Virus- Bi shPLCβ1, Bi shSCR) X (Cue– +Odor, -Odor)] for number of c-Fos positive cells in the OC revealed no significant main effect or interaction (*p*s > .05) (Bi shPLCβ1 +Odor, N = 3, n = 6; Bi shPLCβ1 -Odor, N = 3, n = 6; Bi shSCR +Odor, N = 3, n = 6; Bi shSCR -Odor, N = 3, n = 6). All values are Mean ± SEM. **p* < .05; ***p* < .01; ****p* < .001.

Reference

Kim, S. W., M. Seo, D. S. Kim, M. Kang, Y. S. Kim, H. Y. Koh and H. S. Shin (2015). "Knockdown of phospholipase C-beta1 in the medial prefrontal cortex of male mice impairs working memory among multiple schizophrenia endophenotypes." J Psychiatry Neurosci **40**(2): 78-88.
